# Effects of negative dietary cation–anion difference and calcidiol supplementation in transition diets fed to sows on piglet survival, piglet weight, and sow metabolism

**DOI:** 10.1093/jas/skae027

**Published:** 2024-01-29

**Authors:** Alice Caroline Weaver, Thomas Craig Braun, Jeffrey Allan Braun, Helen Marie Golder, Elliot Block, Ian John Lean

**Affiliations:** South Australian Research and Development Institute, Rosedale, SA 5350, Australia; Myora Farm, Glenburnie, SA 5291, Australia; Myora Farm, Glenburnie, SA 5291, Australia; Scibus, Camden, NSW 2570, Australia; Arm & Hammer Animal Nutrition, Princeton, NJ 08543, USA; South Australian Research and Development Institute, Rosedale, SA 5350, Australia; Scibus, Camden, NSW 2570, Australia

**Keywords:** calcidiol, dietary cation–anion difference, stillbirth, transition diet

## Abstract

Diets that provide a negative dietary anion cation difference (**DCAD**) and supplement with a vitamin D metabolite 25-OH-D_3_ (calcidiol) may increase calcium availability at parturition, and enhance piglet survival and performance. This factorial study assessed the effects of DCAD, calcidiol (50 µg/kg), and parity (parity 1 or >1) and their interactions. Large White and Landrace sows (*n* = 328), parity 1 to 8 were randomly allocated in blocks to treatment diets from day 103 of gestation until day 3 postfarrow: 1) negative DCAD without calcidiol (negative DCAD + no CA), *n* = 84, 2) negative DCAD with calcidiol (negative DCAD + CA) *n* = 84, 3) positive DCAD without calcidiol (negative DCAD + no CA), *n* = 81, and 4) positive DCAD with calcidiol (positive DCAD + CA), *n* = 79. Negative DCAD diets were acidified with an anionic feed (2 kg/t) and magnesium sulfate (2 kg/t). All treatment diets contained cholecalciferol at 1,000 IU/kg. Dry sow diets contained 14.8% crude protein (**CP**), 5.4% crude fiber (**CF**), 0.8% Ca, and 83 mEq/kg DCAD. Treatment diets 1 and 2 contained 17.5% CP, 7.3% CF, 0.8% Ca, and −2 mEq/kg DCAD. Treatment diets 3 and 4 contained 17.4% CP, 7.4% CF, 0.8% Ca, and 68 mEq/kg DCAD. Before farrowing, all negative DCAD sows had lower urine pH than all sows fed a positive DCAD (5.66 ± 0.05 and 6.29 ± 0.05, respectively; *P* < 0.01); urinary pH was acidified for both DCAD treatments indicating metabolic acidification. The percentage of sows with stillborn piglets was not affected by DCAD, calcidiol, or parity alone but sows fed the negative DCAD + CA diet had a 28% reduction in odds of stillbirth compared to the negative DCAD + no CA diet and even lesser odds to the positive DCAD + CA diet. At day 1 after farrowing, blood gas, and mineral and metabolite concentrations were consistent with feeding a negative DCAD diet and that negative DCAD diets influence energy metabolism, as indicated by increased glucose, cholesterol, and osteocalcin concentrations and reduced nonesterified free fatty acids and 3-hydroxybutyrate concentrations. In the subsequent litter, total piglets born and born alive (14.7 ± 0.3 and 13.8 ± 0.3 piglets, respectively; *P* = 0.029) was greater for positive DCAD diets compared to negative DCAD diets; and there was an interaction between DCAD, calcidiol, and parity (*P* = 0.002). Feeding a negative DCAD diet influenced stillbirth, subsequent litter size, and metabolic responses at farrowing. More studies are needed to define optimal diets prefarrowing for sows.

## Teaser

This study found that feeding a negative dietary cation–anion difference (**DCAD**) diet before parturition reduced stillbirth and urine pH and altered metabolism at farrowing. The negative DCAD diets had fewer piglets born in a subsequent litter.

## Introduction

Maximizing the number of healthy, robust piglets weaned per sow is a key objective for most swine producers. Increasing litter size can increase meat yield; however, large litter sizes can increase piglet mortality with ~15% to 20% of piglets dying either during the farrowing process or in early lactation (Farmer and Edwards, 2022). Reducing piglet loss prior to weaning is key to increasing weaned litter size.

Sows experience profound physiological and environmental changes in the transition period from gestation, through parturition and into lactation. The transition period is relatively short for sows and has been defined as the last 10 d of gestation and the first 10 d of lactation (Theil, 2015). During this transition period, sows change from an anabolic to a catabolic state, have dietary changes, move from group housing to individual housing, undergo parturition, produce and secrete colostrum, and initiate and maintain milk production. Current feeding practices may not adequately address these changes and there is evidence that the transition period is a strong determinant of successful lactation (Theil, 2015).

In the dairy industry, it is common for producers to feed their cows a diet with a negative dietary cation–anion difference (**DCAD**) during the transition period to reduce the risk of hypocalcemia (Houe et al., 2001). Dietary DCAD reflects the difference between milliequivalents of the principal cations (potassium, K and sodium, Na) and the principal anions (chloride, Cl; sulfur, S). A negative DCAD diet is commonly achieved by including acidogenic protein meals in the ration. Negative DCAD transition diets increase milk production and reduce the incidences of mastitis, metritis, dystocia, and retained placenta in cattle (Lean et al., 2019). Blim et al. (2022) found that sows with low total blood Ca had a greater risk of dystocia. A negative DCAD diet in late gestation and early lactation increased Ca mobilization from the skeleton (Darriet et al., 2017), tended to reduce stillborn piglets (Henman et al., 2023), increased subsequent litter size (Roux et al., 2008; Henman et al., 2023), and increased feed intake in lactation (Henman et al., 2023). If feed intake improves milk quality and quantity, this may increase the weaning weight of piglets and reduce age-to-slaughter weight (Wolter and Ellis, 2001). The risk and incidence of hypocalcemia in sows is less understood than in the dairy industry. Darriet et al. (2017) hypothesized sows are at risk of hypocalcemic disorders due to the link between increased milk production and an increase in unexplained sow periparturient mortality. There is evidence in mice, humans, and cattle that metabolic effects, including energy metabolism, are orchestrated by osteocalcin produced by mature osteoblasts in the bone by upregulating metabolism in response to periods of high metabolic demand such as lactation (Lean et al., 2014). Consequently, metabolic acidosis may influence the skeleton and play an essential role in energy metabolism (Lee et al., 2007; Lean et al., 2014) reflecting a crucial need to integrate homeorhetic changes that are required to upregulate glucose and fat metabolism in response to the demands of lactation (Lee et al., 2007; Lean et al., 2014). Understanding the physiology of transition in pigs may provide nutritional strategies that allow the sow to prepare and cope with the rapid and substantial increase in metabolic demand around farrowing.

Dietary vitamin D is also important to ensure Ca uptake as many sows are housed indoors, raising the potential for vitamin D insufficiency, notwithstanding cholecalciferol supplementation. Benefits of including calcidiol in sow rations have been observed (Lauridsen et al., 2010; Weber et al., 2014; Meuter et al., 2016; Sørensen and Nielsen, 2016) with colostrum (Weber et al., 2014) and milk production increased (Weber et al., 2014; Meuter et al., 2016). Inclusion of calcidiol in diets has also been associated with fewer farrowing complications (Meuter et al., 2016), increased piglet birth weight (Weber et al., 2014; Sørensen and Nielsen, 2016), reduced incidence of fever (Meuter et al., 2016), and decreased number of stillborn piglets when sows were supplemented at levels of 1,400 IU/d or higher (Lauridsen et al., 2010).

Information is lacking, however, on the optimal DCAD content in the diet and the optimal time to feed a positive DCAD diet after farrowing. Further, there are few data on the effect of implementing a negative DCAD transition feeding strategy on production responses including farrowing performance, lactation performance, sow feed intake, pre- and postweaning piglet growth, and subsequent sow fertility.

The aim of this experiment was to evaluate the performance, health, and reproduction of commercial sows fed different diets over the transition period (~14 d prefarrowing until 3 d postfarrowing). Specifically, this study aimed to investigate the effect of transition rations with either a positive or negative DCAD diet alone or in combination with calcidiol. This project had three objectives 1) to determine if feeding a negative DCAD transition diet from late in gestation to early lactation will improve production outcomes, 2) To evaluate whether there is evidence that the skeleton regulates energy metabolism in the pig as indicated by changes in blood metabolites, as it does in other species, and 3) to determine if there is a positive interaction of both DCAD and the inclusion of calcidiol in sow transition diets.

## Materials and Methods

This study was approved by the Department of Primary Industries and Regions South Australia Animal Ethics Committee (#10/19) and was conducted in accordance with the Australian Code for the Care and Use of Animals for Scientific Purposes. All animal work was conducted at Myora Farm’s Breeder and Grower Facility, Glenburnie, South Australia.

### Experimental design and diets

This study was conducted from March 2020 to September 2020 over 10 weekly farrowing batches. A total of 328 purebred Landrace (*n* = 160) or Large White (*n* = 168) sows (Myora Genetics, Glenburnie, South Australia, Australia) were enrolled in the experiment, and selected sows were either primiparous (*n* = 99) or multiparous (*n* = 229; average parity = 2.59 ± 1.51; parity range = 1 to 8). Sows were randomly allocated in blocks using the *ralloc* function in Stata version 14.1 (StataCorp LP, College Station, TX) to one of four treatment diets based on their breed and their status of being primiparous or multiparous. Treatment diets were 1) negative DCAD without calcidiol (CA; negative DCAD + no CA; *n* = 84); 2) negative DCAD with calcidiol (negative DCAD + CA; *n* = 83); 3) positive DCAD with no calcidiol (positive DCAD + no CA; *n* = 81); and 4) positive DCAD with calcidiol (positive DCAD + CA; *n* = 79). Negative DCAD diets were achieved by the inclusion of 2 kg/t of both BioChlor (an acidogenic protein meal; Arm and Hammer Animal Nutrition, Princeton, NJ), and magnesium sulfate. Positive DCAD diets were achieved by the inclusion of sodium bicarbonate at 4 kg/t. Treatment diets were fed from day 104 of gestation to 3 d postfarrowing. Calcidiol (Rovimix HyD; DSM Nutritional Products, Basel, Switzerland) was included at 1.5 kg/t of feed to deliver 50 µg/kg of finished feed. All diets contained 1,000 IU/kg of cholecalciferol. Diet formulations are outlined in Table 1, and nutrient specifications of each diet are shown in Table 2.

**Table 1.** Composition of dry sow, negative DCAD, and positive DCAD transition diets (±calcidiol) fed during the trial (kg per t)

Feed ingredient, kg	Dry sow	Lactating sow	Negative DCAD	Negative DCAD + calcidiol	Positive DCAD	Positive DCAD + calcidiol
Barley	548	187	255	255	255	255
Wheat	200	400	248	246	248	246
Lupins	75	150	120	120	120	120
Full fat soya	0	25	30	30	30	30
Canola meal expeller	35	20	30	30	30	30
Meat and bone meal	20	35	12	12	12	12
Soybean extract	20	30	0	0	0	0
Fishmeal	35	50	40	40	40	40
Lucerne chaff	0	0	70	70	70	70
Oat hulls	40	0	0	0	0	0
Bran	0	28	80	80	80	80
Sugar beet pulp pellets	0	0	35	35	35	35
Flax seed oil	5	25	34	34	34	34
Lysine HCl	1.2	1.8	1.1	1.1	1.1	1.1
MHA methionine	1.2	1.9	0.9	0.9	0.9	0.9
Threonine	0.9	1.5	0.9	0.9	0.9	0.9
Tryptophan	0.2	0.7	0.4	0.4	0.4	0.4
Valine	0.0	1.8	1.0	1.0	1.0	1.0
l-Arginine	0.5	0.0	0.5	0.5	0.5	0.5
Dicalcium phosphate	3.0	4.5	0.0	0.0	0.0	0.0
Salt	3.0	0.9	4.1	4.1	4.1	4.1
Sodium bicarbonate	6.0	10.5	0.0	0.0	4.0	4.0
Potassium carbonate	1.0	3.0	0.0	0.0	0.0	0.0
BioChlor^1^	0.0	0.0	2.0	2.0	0.0	0.0
Magnesium sulfate	0.0	0.0	2.0	2.0	0.0	0.0
Rovimix HyD^2^	0.0	0.0	0.0	1.5	0.0	1.5
Yang^3^	0.0	0.0	0.4	0.4	0.4	0.4
Hilyses prebiotic^4^	0.0	8.3	10.0	10.0	10.0	10.0
Levucell SB10ME^5^	0.0	0.0	0.1	0.1	0.1	0.1
Betaine 96^6^	0.0	2.0	2.0	2.0	2.0	2.0
Opticell^7^	0.0	8.0	15.0	15.0	15.0	15.0
Mastersorb Gold MycoBind^8^	1.0	1.0	1.5	1.5	1.5	1.5
Activo^9^	0.10	0.15	0.25	0.25	0.25	0.25
Vitamin mineral premix^10^^, 11^	3.5	3.5	3.5	3.5	3.5	3.5
Choline Chloride 60%	0.5	0.5	0.5	0.5	0.5	0.5

^1^BioChlor (an acidogenic protein meal; Arm and Hammer Animal Nutrition, Princeton, NJ).

^2^Rovimix HyD (Division of Animal Nutrition and Health, DSM Nutritional Products LLC, Parsippany, NJ).

^3^Yang (inactivated yeast fractions; Lallemand Animal Nutrition, Canada).

^4^HiLysis (hydrolyzed yeast; YorkAg Products Inc, York, PA).

^5^Levucell (active yeast; Lallemand Animal Nutrition, Canada).

^6^VistaBet (ABVista, Wiltshire, UK).

^7^OptiCell (fiber source; Agromed Austria GmbH, Austria).

^8^Mastersorb Gold MycoBind (mycotoxin binder; EW Nutrition, Adel, IA).

^9^Activo Xtract 6930 (phytochemicals; EW Nutrition, Adel, IA).

^10^Dry sow premix (vitamin A 15.0000 MIU, vitamin D3 1.0000 MIU, vitamin E 250.0000 G, vitamin K3 4.0000 G, vitamin B1 3.00 G, vitamin B2 10.00 G, vitamin B6 5.00 G, vitamin B12 0.05 G, Biotin 0.80 G).

^11^Transition and lactation premix (vitamin A 15.0000 MIU, vitamin D3 1.0000 MIU, vitamin E 250.0000 G, vitamin K3 4.0000 G, vitamin B1 3.00 G, vitamin B2 10.00 G, vitamin B6 5.00 G, vitamin B12 0.05 G, Biotin 0.80 G).

**Table 2.** Nutrient analyses (calculated) of diets fed during the experiment

Nutrient	Dry sow	Lactating sow	Negative DCAD (∓calcidiol)	Positive DCAD (∓calcidiol)
Dry matter, %	90.2	90.6	90.5	90.5
Digestible energy, MJ/kg	13.8	14.5	13.8	13.8
Crude protein, %	14.8	19.5	17.5	17.4
Crude fiber, %	5.37	4.94	7.36	7.35
NDF, %	15.2	13.0	18.3	18.2
ADF, %	6.52	6.24	9.11	9.10
Lysine (total), %	0.67	0.95	0.90	0.89
Digestible Lysine:energy, g/MJ	0.51	0.69	0.59	0.59
Calcium, %	0.81	1.15	0.83	0.83
Phosphorous, %	0.68	0.64	0.60	0.60
Magnesium, g/kg	1.44	1.57	1.92	1.69
Potassium, %	0.62	0.79	0.67	0.67
Sodium, %	0.35	0.41	0.34	0.34
Chloride, %	0.40	0.29	0.50	0.48
Salt, %	0.66	0.49	0.80	0.79
Sulphur, %	0.18	0.20	0.22	0.19
DCAD, mEq/kg	83.4	178	-2.1	68.4
Dietary electrolyte balance	198	301	136	186

### Animal housing and management

Sows were previously mated by artificial insemination with either single sire Landrace or Large White fresh extended semen. Thus, sows produced either purebred or crossbred litters. Sows were pregnancy scanned at ~28 d postmating via trans-abdominal ultrasonography (ImaGo.S, ECM International Inc, Angoulême, Charente, France). During gestation, sows were housed in groups of up to 35 animals on straw and sawdust bedding. Sows were fed a dry sow ration in open full-body stalls twice daily at ~0800 and 1500 hours via an automatic trickle feed system (Hotraco Agri, Hegelsom, the Netherlands). On day 104 of gestation, sows started to receive their experimental rations in the dry sow shed. At feeding time, all sows were locked in individual feeding stalls, and sows were hand-fed their diets (one full feed scoop; ~2.5 kg per sow). After ~1 h, any feed residuals were recorded—visually appraised, and classified as either 1) ate full ration, 2) ate three-fourths of the ration, (3) ate two-fourths of the ration, (4) ate one-fourth of the ration, or 5) ate none of the rations—and removed from the feed bowl, and sows were released from the feeding stalls.

On day 109 of gestation, sows were moved from the dry sow group housing to individual farrowing crates where they remained until weaning. Sows had ad libitum access to fresh drinking water. Farrowing rooms were temperature controlled (17 to 21 °C, depending on the physiological state) and each farrowing crate had a heat mat provided for an additional heat source for the piglets. Sows were fed three times daily at ~0730, 1230, and 1530 hours. Before farrowing, sows received 4 kg/d (1.33 kg at each meal) of the transition diets. After farrowing, feed bowls were checked every morning by a piggery attendant, and depending on the estimated amount of feed left in the bowl, the feed level was either increased, decreased, or remained the same. The amount of feed given each day was recorded. Feed intake in lactation increased from 3.5 kg on day 1 postfarrowing to a maximum of 13 kg by weaning. On day 4 postfarrowing, sows ceased their experimental transition diets and were fed a standard lactating sow ration until weaning.

Litters were processed within 24 h of farrowing (approximate range 2 to 16 h). Sows that were farrowed overnight (from 1600 hours) were processed the following morning. Sows that completed farrowing during piggery attendant working hours, were processed in the afternoon at least 2 h after the placenta had been passed. At processing, piglets all received a litter-specific ear-notch number, piglet sex was recorded, piglets had an individual radio frequency identification (**RFID**) ear tag inserted, teeth were clipped, and a 1-mL iron injection was given intramuscularly (Feron 200 + B12, 200 mg/mL iron dextran and 40 µg/mL cyanocobalamin; Bayer Healthcare, Pymble, NSW, Australia). As this experiment was conducted on a commercial farm, some cross-fostering was required to minimize unnecessary piglet losses and whenever possible, cross-fostering occurred within treatment. Cross-fostering occurred if litter size exceeded the number of available functional teats and piglets that were failing to thrive were removed as required. Additional piglets from outside the experiment were fostered onto sows in the experiment only if necessary and if teat capacity allowed.

At 4 d of age, all piglets were tail-docked and received a 2-mL oral drench of coccidiocide (Baycox, Bayer, Pymble, NSW, Australia) and a 2-mL injection of *Mycoplasma hyopneumoniae* vaccination (RespiSure ONE, Pfizer Animal Health, West Ride, NSW, Australia). At 21 d of age, all piglets received a second injection of RespiSure and 1 mL IM CircoFLEX (Porcine Circovirus associated disease vaccine, Boehringer Ingleheim Vetmedica, Berkshire, UK). Piglets were weaned at 27.5 ± 0.2 d of age and transported via trailer to the grower facility. Sows were moved to individual weaning pens on concrete slatted floors. Twice daily estrus checks were performed by walking a mature boar in front of each pen and performing a back pressure test to check for standing estrus. Any sows that exhibited a standing reflex were inseminated twice 12 h apart.

### Body composition

Upon entry to the farrowing house, on day 21 of lactation, and at weaning, sows were weighed, and backfat at the P2 position was measured using an ultrasound machine and sector probe (ImaGo.S, ECM International Inc). Additionally, a subset of 20 sows per treatment were weighed and P2 backfat was recorded in 254 sows on day 1 post-parturition.

### Urine pH

To determine whether metabolic acidosis was being achieved in response to the different DCAD diets prior to farrowing, sow urine pH was measured. Urine was collected at 0700 hours daily from individual pregnant sows in the farrowing house, ~30 min before the morning feed. From 2 d post entry to the farrowing house, all sows were encouraged to stand, and ~20 mL of urine was collected mid-stream from any sows that subsequently urinated. Once a sow had two daily urine samples collected prior to farrowing, no further attempts were made to collect a daily sample. Urine samples were immediately tested for pH using a handheld pH meter (LAQUAtwin B-712, HORIBA Ltd., Kyoto, Japan). Statistical analysis was conducted on samples obtained from 171 sows on the day before farrowing. From 10 d postfarrowing, daily urine samples were collected from as many sows as possible using the same method as described above.

### Blood sample collection and blood gas analysis

Blood samples were collected at entry to the farrowing house, and on days 1 and 21 postpartum from the same subset of 20 sows per treatment that were weighed on day 1 postpartum. Sows were restrained by a snout snare and blood samples were collected via jugular venipuncture using an 18 g 1.5” vacutainer needle and 9 mL Lithium Heparin vacutainer tubes (BD Vacutainer, BD, Belliver Industrial Estate, Plymouth, UK). For day 1 postfarrowing samples only, 100 µL of blood was immediately analyzed for blood chemistry, metabolites, and gases using an EPOC blood analysis system (Siemens Healthineers, Ottawa, Canada). Blood samples were then placed on ice, transported to the laboratory, and processed within 2 h of collection. Blood samples were centrifuged at 1512 × g for 20 min at room temperature and plasma was stored in triplicate at −20 °C until analysis.

### Blood sample analysis

Aliquots of frozen plasma were transported to The University of Sydney Veterinary Pathology Diagnostic Services (Camden, NSW, Australia) for 3-hydroxybutyrate (**BHB**), Ca, cholesterol, Mg, nonesterified free fatty acids (**NEFA**) and P analysis; and The University of Adelaide Research Assay Facility (Adelaide, SA, Australia) for leptin and insulin analysis. A final aliquot of plasma was analyzed for osteocalcin concentration at the South Australian Research and Development Institute’s Turretfield Research Centre (Rosedale, SA, Australia). Plasma Ca, cholesterol, and P were measured in singlicate using Thermo Fisher Scientific Oy (Vantaa, Finland) kits 981367/981772, 981813/981812, and 981890/981891, respectively, according to the manufacturer’s protocols on a Konelab 20XTi analyzer (Thermo Fisher Scientific Oy). Plasma BHB, Mg, and NEFA were measured in singlicate using Randox Laboratories (Crumlin, United Kingdom) kits RB 1007, MG 3880, and FA 115, respectively, according to the manufacturer’s protocols.

Plasma leptin and insulin were measured in duplicate using the radioimmunoassay (**RIA**) kits Multi-Species Leptin RIA Kit (cat. no. XL-85K, Merck Millipore, Darmstadt, Germany) and Porcine Insulin RIA Kit (cat. no. PI-12K, Merck Millipore), respectively. The leptin and insulin assays were performed according to the manufacturer’s instructions, including standard and sample tubes. Plasma osteocalcin was measured in duplicate using a commercially available enzyme-linked immunosorbent assay (N-MID Osteocalcin Enzyme-Linked Immunosorbent Assay, IDS Immunodiagnostic Systems GmbH, Frankfurt am Main, Germany). Inter-assay CVs < 15% and intra-assay CVs < 10% were considered acceptable for all assays.

### Farrowing characteristics and piglet weights

The number of piglets born alive, dead, and mummified was recorded for each sow. All sows that farrowed during the day were supervised by piggery attendants with sows checked every 30 to 60 min. No piggery attendants were present from 1700 to 0600 hours. An internal examination was performed if a piglet was stillborn, or if an inter-piglet interval exceeded 45 min. The number of internal examinations and the number of piglets pulled alive and dead were recorded.

Piglets were individually weighed at litter processing and at 3 and 21 d of age. Any fostered piglets (including those with no RFID tags) were also weighed to calculate the total litter weights for each sow.

### Colostrum and milk collection and analysis

For sows that were farrowed during the day, a presuckle colostrum sample (5 to 10 mL) was collected when possible across all teats after the birth of the first piglet. Colostrum samples (*n* = 86) were immediately analyzed for total solids content (%) using a digital handheld refractometer (Starr Instruments: Model DBR-1, Dandenong South, VIC, Australia). Colostrum was then frozen at −20 °C until immunoglobulin G (**IgG**) analysis. IgG concentration was determined by a previously validated radial-immuno diffusion assay developed by the University of Adelaide’s Veterinary Diagnostic Laboratory (Roseworthy Campus, Roseworthy, SA, Australia). Methods were utilized by a previous method described by Brougham et al. (2020) where 150 µL of swine antigen, and 0.5, 0.25, 0.125, and 0.063 mg/mL of purified swine IgG were used in place of the ovine antigen and purified ovine IgG, respectively. The colostrum samples were diluted with phosphate-buffered saline to a 1:160 dilution prior to IgG analysis.

At 2 d postweaning, milk samples (50 mL) were collected from a subset of 56 sows only (negative DCAD + no CA, *n* = 17; negative DCAD + CA, *n* = 15; positive DCAD + no CA, *n* = 20; positive DCAD + CA, *n* = 16). Sows were walked to individual stalls and milk was collected and pooled from all functional teats. Following sample collection, sows were moved back to their weaning pen. Milk samples were placed into vials containing a milk preservative and shipped to a commercial milk analysis laboratory (Dairy Express Herd Recording Service, University of New England, Armidale, NSW, Australia) to determine fat, protein, and lactose percentage, and urea, and somatic cell content.

### Fecal consistency score

To determine the degree of constipation, a daily visual fecal consistency score was recorded for all un-farrowed sows in the farrowing house until farrowing had occurred. Fecal scores were recorded each morning prior to feces being scraped from behind the sows and an average fecal consistency score was calculated for each sow. A scoring system of 0 to 5 was used as described by Oliviero et al. (2009) with 0 (absence of feces), 1 (dry and pellet shaped), 2 (between dry and normal), 3 (normal and soft, but firm and well-formed), 4 (between normal and wet, still formed but not firm), and 5 (very wet feces, unformed, and liquid).

### Sow and piglet health and mortalities

Sows and piglets were monitored daily for any signs of ill health by trained piggery attendants. Any incidence of mastitis, udder edema, udder engorgement, vaginal discharge, or retained piglets/placenta was recorded as was any medication administered.

Any sow or piglet deaths were recorded including date and cause of death, and piglets were euthanized if any deformities or health issues negatively impacted quality of life. Common causes of piglet mortality were stillborn, overlay, ill thrift, diarrhea, deformity, and low birth weight. Death causes with a low prevalence were incorporated into the category “other” to enable analysis.

### Sow survival and general censoring

Sows were terminated from their treatment group on the date they were mated postweaning, culled, died, or reached day 30 postweaning, whichever occurred first. Sows that died or were culled were terminated from the weaning data on the date they were removed from the herd. These sows were censored from the survival and reproduction data at that point.

### Subsequent reproduction

Weaning to estrus length was recorded in days and subsequent reproduction was also recorded. Measures included conception rate, pregnancy rate, litter size, and number of piglets born alive and dead.

### Sample size estimations

The unit of measurement used for sample size determination in this study was the piglet and the outcome of interest was the stillborn piglet. We estimated an increase in the number of piglets born alive by 0.2 per litter (effect size = 0.25) with the number of piglets per litter per treatment group being estimated from farm data at *n* = 12. The SD was 1.40 and the mean number of stillborn was 1.07 based on 5,327 previous farrowings. This provided a power of 0.81 with an α of 0.05. The estimates were made using *rdpower* (StataCorp LP, version 14.1) with 70 clusters (sows) at level 2, 12 piglets per litter, and an intraclass correlation of 0.2 per treatment. The total piglet number estimated was 1,680 per treatment.

### Statistical analysis

All statistical analysis was conducted using Stata version 17 (StataCorp LLC, College Station, TX). Initial evaluation included tabulation of data by categorical outcomes and visual and statistical appraisal of continuous variables for normality of distribution and the need to transform data to achieve a normal distribution of residuals. For all sow data, the unit of interest was the sow. For all piglet individual weights, weight gains, and survivals, the piglet was used as the unit of measurement. Piglets fostered outside of their birth sow treatment (*n* = 1,194) were excluded from individual piglet weight analysis; however, they were included in the total litter weights for each sow to assess sow milk production. Data were analyzed to evaluate the effects of DCAD, calcidiol supplementation, parity, and their interactions in this factorial analysis. Each variable was evaluated for covariable effects that could influence results, such as sire of piglets, days on feed, breed of sow, and farrow house entry weight. These covariables differed for different outcomes and were tested in models as indicated in Tables 3 to 14. The nonsignificant covariables were removed from the model by backward stepping. Significance was at α < 0.05 and a tendency was noted at *P* < 0.1.

**Table 3.** Sow liveweight, and liveweight change over the experimental period for sows that received either a negative or positive DCAD transition diet and with or without calcidiol (CA). Values for treatments are marginal means ± SE

Item	*n*	Negative DCAD		Positive DCAD		Parity		*P* value				
		No CA	CA	No CA	CA	Primiparous	Multiparous	DCAD	Calcidiol	DCAD × calcidiol	Parity	DCAD × calcidiol × parity
Liveweight, kg												
FH entry^1^	318	337.5 ± 3.2	336.3 ± 3.1^a^	349.0 ± 3.3	342.1 ± 3.3	310.5 ± 2.9^a^	354.7 ± 2.0^b^	0.010	0.223	0.349	0.001	0.807
Day 21^2^	292	303.0 ± 1.5	301.0 ± 1.5	298.7 ± 1.6	301.9 ± 1.6	298.7 ± 1.6	302.3 ± 1.0	0.147	0.398	0.155	0.073	0.713
Weaning^2^	279	296.2 ± 2.0^a^	292.2 ± 1.9^a,b^	289.9 ± 1.9^b^	293.5 ± 2.0^a,b^	290.8 ± 2.1	293.7 ± 1.3	0.169	0.974	0.036	0.234	0.749
Weight change, kg												
FH entry to day 21^3^	289	−37.5 ± 1.5	−39.4 ± 1.5	−42.2 ± 1.6	−38.6 ± 1.6	−40.5 ± 1.4	−38.9 ± 1.0	0.092	0.325	0.136	0.340	0.695
FH entry to weaning^4^	277	−44.4 ± 1.9	−48.5 ± 1.8	−50.2 ± 1.8	−47.7 ± 1.9	−49.7 ± 2.0	−46.9 ± 1.2	0.144	0.853	0.061	0.235	0.805

^1^Covariable was breed (Not significant *P* > 0.05).

^2^Covariables were breed (*P* < 0.001) and farrow house entry weight (*P* < 0.001).

^3^Covariable was breed (*P* < 0.001).

^4^Covariables were breed (*P* < 0.001), farrow house entry weight (*P* < 0.007), and sire of piglet breed (*P* = 0.042).

^a,b^Different superscripts within a row and within treatment or parity indicate pairwise comparisons *P* < 0.05.

FH, farrow house.

Due to the extensive and differing nature of the observed outcomes and differences in the unit of measurement, several different statistical approaches were used. For continuous data for sows (sow weight, backfat, urine pH, fecal consistency score, blood parameters, milk parameters, and litter characteristics) a mixed model analysis (*mixed*) was conducted with the random effect of sow within the block. For continuous data for piglets (piglet weight and weight gain) a mixed model analysis was conducted with the random effect of piglet with sow within the block. For some count data, such as stillbirth, following initial exploration of the data, a Poisson regression indicated that the data were over-dispersed and use of a negative binomial model (*xtnbreg*) for data analysis was indicated. Lactational incidence data including disease were evaluated using a mixed-effects multi-level model using the *melogit* function that provided an evaluation of the odds of disease. The models included sow within the block as a random effect for sow data or piglet within sow within the block.

Post hoc analyses included analysis of the significance of main effects and interactions that were tested with *contrast*, marginal means were calculated with *margins* and pairwise comparisons were made with *pwcompare*. Covariables that may influence outcomes including days on transition, breed of sire, breed of sow, and weight at farrowing house entry were tested in the models and significant covariables are listed in relevant tables.

## Results and Discussion

### Sow body composition

Sow liveweight is presented in Table 3. At farrowing house entry, despite randomization, sows in the negative DCAD treatments weighed less than positive DCAD sows (*P* < 0.05); however, this difference was not evident at day 21 postpartum nor at weaning when covariables were included in the model. Sows were weighed for the first time when moving from the dry sow shed to the farrowing shed due to logistical limitations; however, diets commenced 5 d prior to weighing. It is unlikely that the 5 d of treatment would have affected liveweight as diets were balanced for energy and there was no observed difference in concentrate feed intake among treatment groups. These differences in weight were accounted for by testing for farrowing house entry weight as a covariable in subsequent models. Primiparous sows weighed less than multiparous sows at the entry to the farrowing house (*P* < 0.05), yet, at weaning there was no difference between parities. There was a significant interaction between DCAD and calcidiol at weaning, whereby negative DCAD + no CA sows were heavier than the positive DCAD + no CA sows, but negative DCAD + CA and positive DCAD + CA were similar in weight to all transition treatment diets. In addition, there was a tendency for a DCAD and calcidiol interaction from farrowing house entry to weaning whereby negative DCAD + no CA sows tended to lose less weight from farrowing house entry to weaning than positive DCAD + no CA sows (*P* = 0.061).

Throughout the trial there was no effect of DCAD, calcidiol, or their interactions on P2 backfat (Supplementary Table S1). However, postfarrowing, multiparous sows had consistently lower backfat than primiparous sows (*P* < 0.05; Supplementary Table S1).

### Feed intake

Feed intake throughout the experiment was not impacted by DCAD, calcidiol, or their interactions with each other (*P* > 0.05). Average daily mean intake for all four treatments from days 1 to 21 postfarrowing was 6.4 ± 0.1 kg. However, multiparous sows ate 200 g more per day than their primiparous counterparts from days 1 to 21 postfarrowing (average intake 6.3 ± 0.1 kg and 6.5 ± 0.1 kg, respectively; *P* = 0.042).

### Urine pH and fecal consistency

On the day before farrowing, negative DCAD-fed sows had a lower urine pH than positive DCAD-fed sows (5.66 ± 0.05 and 6.29 ± 0.05, respectively; *P* < 0.001). Mean urine pH prior to farrowing was unaffected by calcidiol, parity, or their interactions with DCAD (*P* > 0.05). Postfarrowing, urine pH was similar between all four transition treatments (overall mean 7.54 ± 0.12; *P* > 0.05). It is notable that the urinary pH for both negative and positive DCAD treatments indicated metabolic acidification, notwithstanding diet formulation. It is possible that fermentation of barley and wheat which comprised ~50% of the diets generated enough volatile fatty acids to reduce urinary pH (Canh et al., 1997; Zhao et al., 2020). The optimal range for urinary acidification has not been established for pigs prefarrowing and it is possible that the optimum may be greater than 5.69. The optimal urine pH for dairy cows ranges between 5.5 and 6.2 (Caixeta and Omontese, 2021), however, the optimal value varies between breeds and publications. A urine pH below 7.0 has been suggested as suitable. Therefore, further studies in the sow are warranted and could focus on determining the optimal urine pH, diet composition, and DCAD value to reflect an appropriate level of metabolic acidification in the transition period.

Fecal consistency scores were higher in all positive DCAD sows (2.26 ± 0.09) compared to all negative DCAD sows (2.59 ± 0.10; *P* < 0.01) but were both close to a “normal” consistency. Lower fecal consistency scores indicate less constipation. This may reflect the inclusion of magnesium sulfate in the negative DCAD diets (Hou et al., 2014). Constipation during the farrowing process can increase stillbirths by creating a physical obstruction of the birth canal and increasing the pain and discomfort which may alter the hormonal pattern of parturition (Oliviero et al., 2010; Langendijk and Plush, 2019). The fecal consistency score values obtained in the current study differed by less than half a score (0.33, of a 1- to 5-scoring system) and despite significant differences between positive and negative DCAD sows, it is unclear if this difference is physiologically relevant. Olivero et al. (2010) found that constipation scores of 1.9 or lower resulted in longer farrowing duration, however, constipation scores in this study were significantly higher and may not have impacted farrowing. There was a significant interaction between DCAD and calcidiol, whereby negative DCAD + no CA sows had a lower fecal consistency score compared to the other three treatments (negative DCAD + no CA, 2.11 ± 0.09; negative DCAD + CA, 2.40 ± 0.09; positive DCAD + no CA, 2.62 ± 0.10; and positive DCAD + CA, 2.67 ± 0.10; *P* < 0.05). Parity also affected fecal consistency score, as multiparous sows had a lower consistency score compared to primiparous sows (2.29 ± 0.06 and 2.67 ± 0.06 respectively; *P* < 0.01).

### Farrowing and litter characteristics

The number of piglets born in a litter, piglets born alive, and litter birth weight was not affected by DCAD, calcidiol, parity, or their interactions (Table 4). The percentage of stillborn piglets within a litter was not affected by DCAD, calcidiol, or parity alone; however, the negative DCAD + CA-fed sows had a lower percentage of stillborn piglets (*P* = 0.005) than the three other diets (Table 4). There are relatively few studies that have looked at negative DCAD in sow rations, and none that examined the interaction between DCAD and vitamin D source. Effects of DCAD on piglet stillbirth rate vary between studies with some positive outcomes and others finding no effect. Guo et al. (2019) reported no effect on the number of stillborn piglets when sows were fed an acidogenic diet from day 108 of gestation; however, the study did not present mean values and animal numbers were low in the experiment (11 sows per treatment). Similarly, (Roux et al., 2008) found no difference in stillbirths when sows were fed a negative DCAD diet from day 111 of gestation to weaning; however, in the subsequent litter, total born and born alive was increased in sows fed the negative DCAD ration. DeRouchey et al. (2003) looked at decreasing levels of dietary electrolyte balance (**dEB**) and found increased piglet survivability as dEB decreased. Cheng et al. (2015) showed lower dEB throughout gestation increased total born and born alive, but stillbirth percentage was not affected. Plush et al. (2018) supplemented sow diets with 0.29% magnesium sulfate, which did not reduce the proportion of stillborn piglets; however, piglets born to magnesium sulfate-fed sows had improved viability. Piglets were quicker to suckle and had higher day-1 glucose concentrations than the controls. The total farrowing duration was 1.2 h longer in the magnesium sulfate-supplemented sows. The inconsistency of results may be due to several factors including DCAD value of the diets, duration of feeding, and parity of the sows. Also of consideration is the formulation of the diets and the ingredients used. The current study found even positive DCAD diets produced acidification, and we hypothesize that fermentation of the carbohydrate fractions in the diet, especially barley and wheat, may have generated enough volatile fatty acids to reduce urinary pH. A deeper understanding of how different diet compositions can influence acid-base balance in sows regardless of DCAD value is warranted particularly to evaluate the role of acid-base and calcium metabolism in parturition.

**Table 4.** The total number of born piglets, piglets born alive, and litter weights and piglet number per sow throughout the experiment for sows that received either a negative or positive DCAD transition diet and with or without calcidiol (CA). Values for treatments are marginal means ± SE

Item	*n*	Negative DCAD		Positive DCAD		Parity		*P* value				
		No CA	CA	No CA	CA	Primiparous	Multiparous	DCAD	Calcidiol	DCAD × calcidiol	Parity	DCAD × calcidiol × parity
Farrowing	315											
Total born, *n*^1^		13.4 ± 0.4	13.2 ± 0.4	13.9 ± 0.4	13.2 ± 0.4	13.1 ± 0.4	13.5 ± 0.2	0.132	0.375	0.375	0.086	0.514
Born alive, *n*^1^		12.0 ± 0.4	12.3 ± 0.3	12.8 ± 0.4	11.9 ± 0.4	12.2 ± 0.4	12.3 ± 0.2	0.179	0.364	0.063	0.858	0.649
Birth litter weight, kg		22.1 ± 0.5	22.0 ± 0.5	23.1 ± 0.5	21.9 ± 0.5	21.8 ± 0.5	22.5 ± 0.3	0.071	0.233	0.100	0.208	0.113
Day 3 postfarrow	299											
Litter size, *n*^2^		12.8 ± 0.1^ab^	13.0 ± 0.1^a^	13.0 ± 0.1^a^	12.6 ± 0.1^b^	12.9 ± 0.1	12.8 ± 0.1	0.439	0.227	0.006	0.634	0.501
Litter weight, kg^3^		28.5 ± 0.5	29.1 ± 0.5	28.4 ± 0.5	28.7 ± 0.5	29.1 ± 0.5	28.5 ± 0.3	0.894	0.612	0.577	0.237	0.379
Day 21 postfarrow	317											
Litter size, *n*^1^		10.1 ± 0.2	10.3 ± 0.1	10.0 ± 0.2	10.0 ± 0.2	10.2 ± 0.2	10.0 ± 0.1	0.972	0.697	0.671	0.289	0.467
Litter weight, kg^2^		85.3 ± 1.7	86.7 ± 1.6	83.6 ± 1.7	86.7 ± 1.7	84.7 ± 1.8	86.0 ± 1.2	0.917	0.346	0.456	0.579	0.498
Number weaned^4^	317	10.0 ± 0.2	10.3 ± 0.2	10.0 ± 0.2	10.0 ± 0.2	10.2 ± 0.2	10.0 ± 0.1	0.948	0.461	0.713	0.203	0.125

^1^Covariable was breed of sow (*P* < 0.05).

^2^Covariable was farrow house entry weight (*P* < 0.05).

^3^Covariables were breed of sow (*P* < 0.05) and days on diet (*P* < 0.001).

^4^Covariables were breed of sow (*P* < 0.05) and farrow house entry weight (*P* < 0.001).

^a,b^Different superscripts within a row and within treatment indicate pairwise comparisons at *P* < 0.05.

While vitamin D requirements are known for gestation and lactation, there is no information on the requirements specifically during parturition (van den Bosch et al., 2023). Calcidiol as an alternative to vitamin D_3_ has been studied in sow diets as it is more bioavailable, efficiently absorbed, bypasses hepatic metabolism, and is three to five times more potent than vitamin D_3_ (Hasan et al., 2023). Few studies have investigated the effect of prepartum calcidiol supplementation on farrowing and lactation outcomes. Apart from one study by Upadhaya et al. (2021) which found an increased number of piglets born alive, overall, vitamin D source had minimal effects on stillbirths (Weber et al., 2014; Zhang et al., 2019; Wang et al., 2020; Upadhaya et al., 2021). Differences among studies could reflect calcidiol inclusion rate, duration of supplementation, and litter size. Further investigations are warranted and should determine the optimal inclusion rate and supplementation period prefarrowing. It would also be worthwhile to investigate calcidiol supplementation on farrowing time and ease, uterine contractility, milk initiation, and milk production.

Despite a significantly smaller litter size for the positive DCAD + CA treatment at day 3, by day 21 of lactation, there was no effect of DCAD, calcidiol, parity, or their interactions on litter characteristics and all sows weaned similar numbers of piglets (Table 4). As this study was conducted on a commercial operation, fostering piglets was required to minimize piglet mortalities. Efforts were made to minimize the amount of fostering, and to foster within treatment groups; however, total piglet movement was ~20%. While effects on stillbirth rate could be a direct effect of the diet fed before farrowing, effects on piglet or litter growth may have been masked by the movement of piglets both on and off a litter. We consider this to be an acceptable outcome as commercial practice would reflect this management decision to foster, hence results obtained are commercially applicable and meaningful to pig producers.

Individual piglet weight at birth, day 3, 7, or 21 did not differ in response to DCAD, calcidiol, or their interaction (Table 5; *P* > 0.05). It is noted that the time of litter processing relative to farrowing may affect the accuracy of piglet birth weight measurement, however, this is unlikely to have impacted the trial outcomes as there was no difference in the number of sows that farrowed outside of piggery working hours among treatments. Parity had no impact on piglet weight at birth or 3 d of age; however, by 21 d of age, piglets born to multiparous sows were 290 g heavier than piglets born to primiparous sows (Table 5; *P* < 0.05). Piglets born to multiparous sows grew 13 g more per day from birth to day 21 (Table 5; *P* = 0.001). There was significant interaction between DCAD, calcidiol, and parity for birth weight whereby piglets born to primiparous sows that received a positive DCAD + no CA diet were heavier than piglets born to primiparous sows that received a negative DCAD + no CA diet and piglets born to multiparous sows that received a positive DCAD + no CA diet (1.74 ± 0.04, 1.61 ± 0.04, and 1.63 ± 0.03 kg, respectively; *P* < 0.05). Piglet average daily weight gain from birth to day 21 was not impacted by DCAD, calcidiol, or their interaction.

**Table 5.** Piglet weight, P2 back fat and average daily gain from sows that received either a negative or positive DCAD transition diet and with or without calcidiol (CA). Values for treatments are marginal means ± SE

Item	*n*	Negative DCAD		Positive DCAD		Parity		*P* value				
		No CA	CA	No CA	CA	Primiparous	Multiparous	DCAD	Calcidiol	DCAD × calcidiol	Parity	DCAD × calcidiol × parity
Piglet weight, kg												
Birth^1^	5,149	1.67 ± 0.02	1.66 ± 0.02	1.67 ± 0.02	1.64 ± 0.02	1.67 ± 0.02	1.66 ± 0.02	0.731	0.303	0.269	0.778	0.047
Day 3^1^	4,096	2.23 ± 0.03	2.23 ± 0.03	2.22 ± 0.03	2.23 ± 0.03	2.23 ± 0.03	2.23 ± 0.02	0.333	0.641	0.872	0.901	0.336
Day 21^2^	3,615	8.41 ± 0.09	8.39 ± 0.08	8.50 ± 0.09	8.51 ± 0.09	8.25 ± 0.08^a^	8.55 ± 0.06^b^	0.343	0.794	0.976	0.001	0.443
Piglet ADG, g/d												
Birth to day 21^3^	3,615	316 ± 4	316 ± 4	319 ± 4	323 ± 4	309 ± 3^a^	322 ± 2^b^	0.352	0.973	0.656	0.001	0.704

^1^Covariables were breed of sow (*P* < 0.001) and litter size (*P* < 0.001).

^2^Covariable was litter size (*P* < 0.001).

^3^Covariable was litter size (*P* < 0.001).

^a,b^Different superscripts within a row and within treatment indicate pairwise comparisons at *P* < 0.05.

The lack of difference in weaning weight for the piglets is consistent with the very similar bodyweights for sows, although the positive DCAD sows lost more body weight from farrow house entry to weaning than the negative DCAD (Table 3). Calcidiol fed in the transition period had no impact on piglet weaning weights which is similar to Weber et al. (2014) but differs from Wang et al. (2020) and Zhou et al. (2017) who found calcidiol increased piglet growth rate. Differences among study results may be due to the period of feeding and time of sampling.

Table 6 shows little difference among groups in neonatal health of piglets except for a significant reduction in the number of litters with stillborn piglets for the negative DCAD + CA diet compared to the negative DCAD + no CA and the positive DCAD + CA diets. This finding is consistent with the data on the effects of an acidogenic diet (Henman et al., 2023) who found that the relative risk for stillbirth was significantly reduced by 32% and 45% by inclusion of an acidogenic protein meal during gestation and lactation. The mean mummified feti per sow were 0.59 for negative DCAD + no CA, 0.75 for negative DCAD + CA, 0.68 for positive DCAD + no CA, and 0.37 for positive DCAD + CA diets (*P* > 0.05). Sow breed differences existed (*P* < 0.001). There was an effect of the sire of litter (*P* = 0.05), and there was a significant effect of DCAD (*P* < 0.05). There was no effect of calcidiol or parity or any interaction (*P* > 0.05) on mummified feti, when evaluated with negative binomial regression. There was a 36% reduction in the incidence of mummified feti in the positive DCAD sows; however, this was unlikely to be influenced by diet in the last 10 d of gestation as the vast majority of the mummified fetuses were small in size (data not shown) and likely died earlier than day 105 of gestation.

**Table 6.** Percentage and odds ratios ± SE in brackets of mortality and neonatal disorders of litters (*n* = 328) born to sows that received either a negative or positive DCAD transition diet and with or without calcidiol (CA). The unit of interest is the sow litter. Estimated percentages for treated sows. Covariables tested were litter size, sex of piglet, and breed of sow and sire

Mortality type	Treatment				*P* value				
	Negative DCAD		Positive DCAD						
	No CA	CA	No CA	CA	DCAD	Calcidiol	DCAD × calcidiol	Parity	DCAD × calcidiol × parity
Stillborn	9.48 referent^a^	6.10 (0.72 ± 0.19^b^)	7.21 (0.75 ± 0.19^ab^)	9.80 (1.24 ± 0.30^a^)	0.671	0.483	0.020	<0.001	0.910
Ill thrift	1.26 referent	2.00 (1.36 ± 0.58)	1.41 (0.95 ± 0.43)	1.10 (0.80 ± 0.38)	0.361	0.839	0.447	0.309	0.468
Overlay^1^	4.22 referent	4.63 (1.06 ± 0.29)	4.74 (1.03 ± 0.28)	6.17 (1.27 ± 0.34)	0.581	0.491	0.692	0.630	0.995
Low birth weight^2^	1.39 referent	0.47 (0.32 ± 0.20)	0.96 (0.70 ± 0.35)	0.71 (0.49 ± 0.30)	0.981	0.091	0.339	NS	—
Scours^3^	0.28 referent	0.37 (1.29 ± 1.06)	0.48 (1.66 ± 1.32)	0.71 (2.48 ± 0.95)	0.270	0.533	0.892	NS	—
Other	0.90 referent	0.84 (1.08 ± 0.56)	1.21 (1.44 ± 0.72)	1.09 (1.33 ± 0.67)	0.399	0.998	0.816	0.745	0.422

^1^Covariable was breed of sow (*P* = 0.033).

^2^Interactions with parity could not be assessed and sex of piglet was a covariable (*P* = 0.022).

^3^Interactions with parity could not be assessed.

^a,b^Different superscripts within a row and within treatment indicate pairwise comparisons at *P* < 0.05.

### Blood measures in sows

Metabolic measures at farrowing house entry are in Table 7. At entry to the farrowing house and after ~5 d on diets, the main effects of DCAD and calcidiol, and the interaction between the two dietary components had no effect on circulating Ca, P, insulin, or osteocalcin concentrations. However, sows fed negative DCAD transition diets had lower cholesterol and glucose concentrations, but higher concentrations of BHB and Mg compared to sows that received positive DCAD transition diets (significant main effects; Table 7). These results would be consistent with a lower intake of feed at this time. Sows fed calcidiol had higher circulating concentrations of cholesterol, glucose, and leptin, but lower concentrations of NEFA than sows without calcidiol indicating less reliance on mobilized lipids and possibly a better energy balance. Sows fed negative DCAD + no CA had lower concentrations of glucose and higher concentrations of BHB and NEFA than sows that received all other transition diets indicating greater reliance on mobilized lipids. Sows fed the negative DCAD + CA ration had higher concentrations of circulating leptin than the three other transition rations.

**Table 7.** Blood metabolite measures of sows at entry to the farrowing house of sows (*n* = 80) that received either a negative or positive DCAD transition diet and with or without calcidiol (CA). Values for treatments are marginal means ± SE. Breed of sow was tested as a covariable

Item	Negative DCAD		Positive DCAD		Parity		*P* value				
	No CA	CA	No CA	CA	Primiparous	Multiparous	DCAD	Calcidiol	DCAD × calcidiol	Parity	DCAD × calcidiol × parity
Calcium, mmol/L	2.48 ± 0.04	2.55 ± 0.03	2.56 ± 0.03	2.53 ± 0.03	2.55 ± 0.04	2.53 ± 0.02	0.391	0.486	0.085	0.754	0.693
Cholesterol, mmol/L	1.77 ± 0.07	1.97 ± 0.05	1.91 ± 0.06	2.14 ± 0.05	1.99 ± 0.06	1.95 ± 0.04	0.013	0.001	0.960	0.605	0.430
Glucose, mmol/L	3.87 ± 0.12^a^	4.43 ± 0.09^b^	4.45 ± 0.10^b^	4.34 ± 0.09^b^	4.29 ± 0.10	4.30 ± 0.07	0.003	0.010	0.001	0.783	0.477
Phosphate, mmol/L	1.93 ± 0.03	1.94 ± 0.03	1.93 ± 0.03	1.90 ± 0.03	2.00 ± 0.03^a^	1.88 ± 0.02^b^	0.424	0.944	0.450	0.003	0.785
BHB, mmol/L	0.20 ± 0.02^a^	0.11 ± 0.02^b^	0.10 ± 0.02^b^	0.11 ± 0.02^b^	0.14 ± 0.02	0.12 ± 0.01	0.001	0.007	0.001	0.245	0.025
Magnesium, mmol/L	0.83 ± 0.02	0.83 ± 0.01	0.79 ± 0.01	0.78 ± 0.01	0.82 ± 0.02	0.80 ± 0.01	0.005	0.715	0.625	0.379	0.530
NEFA, mmol/L	0.76 ± 0.09^a^	0.35 ± 0.07^b^	0.34 ± 0.07^b^	0.51 ± 0.07^b^	0.57 ± 0.07	0.42 ± 0.05	0.070	0.039	<0.001	0.105	0.260
Insulin, μIU/mL^1^	1.73 ± 0.13	1.96 ± 0.10	1.89 ± 0.11	1.94 ± 0.11	2.06 ± 0.12	1.80 ± 0.07	0.476	0.379	0.392	0.082	0.718
Leptin, ng/mL	6.46 ± 0.93^a^	10.77 ± 0.76^b^	8.68 ± 0.82^a^	7.73 ± 0.83^a^	8.52 ± 1.08	8.42 ± 0.65	0.425	0.017	<0.001	0.952	0.317
Osteocalcin, ng/mL	95.79 ± 9.20	111.26 ± 7.34	95.38 ± 7.99	92.71 ± 7.77	111.61 ± 8.35	92.29 ± 5.33	0.265	0.466	0.305	0.081	0.976

^1^Log transformed.

^a,b^Different superscripts within a row and within treatment indicate pairwise comparisons at *P* < 0.05.

Parity had few effects on circulating blood measures at farrowing house entry except that primiparous sows had a higher P concentration than multiparous sows. There was one significant DCAD, calcidiol, and parity interaction whereby primiparous sows fed a negative DCAD + no CA diet had higher BHB (0.29 ± 0.02 mmol/L) than all other groups (primiparous negative DCAD + CA, 0.11 ± 0.03; primiparous positive DCAD + no CA, 0.09 ± 0.03, primiparous positive DCAD + CA, 0.10 ± 0.02; multiparous negative DCAD + no CA, 0.15 ± 0.02; multiparous negative DCAD + CA, 0.11 ± 0.03; multiparous DCAD + no CA, 0.10 ± 0.02; and multiparous positive DCAD + CA, 0.12 ± 0.02 mmol/L).

The quite large effects of diet on metabolites after 5 d on diet at entry to the farrowing house may reflect, in part, reduced dry matter intake (DMI) of acidogenic diets, as acidogenic diets can reduce DMI in cows (Zimpel et al., 2018). We evaluated feed intake by visual appraisal only and did not weigh the feed given nor the feed refusals which would have increased the accuracy of feed intake data. However, refusals of concentrate fed prior to farrowing house entry were minimal with 95% to 97% of sows consuming their entire ration. This 4 kg of feed was designed to meet the nutritional requirements of the sows. Sows fed negative DCAD diets had lower glucose and higher BHB concentrations which both can reflect reduced DMI. One explanation could be that straw was provided as a bedding substrate which is regularly consumed by sows. There was no way to differentiate the straw intake of individual animals which may have impacted total DMI and hence blood metabolites.

Table 8 presents blood measures on day 1 post-parturition. Sows fed negative DCAD transition diets had lower concentrations of bicarbonate, base excess in the extracellular fluid compartment and blood, higher oxygen partial pressure, and osteocalcin concentration than sows that received positive DCAD rations (*P* < 0.05). These findings in blood gas, and mineral and metabolite concentrations are consistent with the negative DCAD in the diet and the increase in osteocalcin is consistent with the observations in dairy cows (Rodney et al., 2018). DeRouchey et al. (2003) also demonstrated lowering the dEB of lactating sow diets decreased base excess of blood and extracellular fluid, bicarbonate, and partial pressure of carbon dioxide which indicates metabolic acidification following the principle of the Henderson–Hasselback equation. Guo et al. (2019) showed a lower DCAD diet in late gestation and lactation reduced blood and urine pH at day 1 of lactation, but not at day 108 of gestation nor at days 9 and 18 of lactation. We did not see a difference in blood pH despite seeing a significant reduction in urine pH. It is well understood that intracellular and extracellular pH is maintained within a tight physiological range to keep enzymes, transporters, and receptors functioning normally (Guo et al., 2019). Therefore, it is perhaps not surprising that blood pH was similar between positive and negative DCAD sows despite significant differences in urine pH. Interestingly, blood Ca concentrations at farrowing did not differ among treatments in our study. This finding is consistent with Roux et al. (2008) but contrasts with the marked increase in blood Ca concentrations in dairy cows exposed to a negative DCAD diet (Rodney et al., 2018) and in lactating sows (Guo et al., 2019). A lower dEB intake has been associated with increased circulating chlorine (DeRouchey et al. 2003; Cheng et al. 2015) and magnesium (Cheng et al. 2015); however, we did not see an increase in either.

**Table 8.** Blood metabolite measures on day 1 postparturition of sows (*n* = 76) that received either a negative or positive DCAD transition diet and with or without calcidiol (CA). Values for treatments are marginal means ± SE. Covariables tested were weight at farrowing house entry, days on transition diet, and breed of sow

Item	Negative DCAD		Positive DCAD		Parity		*P* value				
	No CA	CA	No CA	CA	Primiparous	Multiparous	DCAD	Calcidiol	DCAD × calcidiol	Parity	DCAD × calcidiol × parity
pH	7.45 ± 0.01	7.45 ± 0.01	7.45 ± 0.01	7.46 ± 0.01	7.45 ± 0.01	7.46 ± 0.01	0.734	0.782	0.448	0.611	0.173
Carbon dioxide partial pressure, mmHg	49.84 ± 1.92	51.02 ± 1.61	52.34 ± 1.60	52.08 ± 1.61	51.71 ± 1.46	51.22 ± 1.08	0.115	0.729	0.496	0.926	0.362
Oxygen partial pressure, mmHg	40.69 ± 2.23^a^	36.77 ± 1.87^ab^	34.34 ± 1.86^b^	32.74 ± 1.87^b^	38.70 ± 1.70^a^	34.43 ± 1.18^b^	0.003	0.032	0.170	0.018	0.009
Bicarbonate, mmol/L	35.07 ± 0.66^a^	35.61 ± 0.56^ab^	36.06 ± 0.55^ab^	36.89 ± 0.56^b^	35.67 ± 0.51	36.09 ± 0.35	0.026	0.147	0.847	0.421	0.910
Base excess (efc), mmol/L	11.17 ± 0.63^a^	11.67 ± 0.53^ab^	12.07 ± 0.52^ab^	13.05 ± 0.53^b^	11.69 ± 0.48	12.22 ± 0.33	0.026	0.094	0.613	0.320	0.741
Oxygen saturation, %	70.05 ± 2.69	69.06 ± 2.25	65.28 ± 2.24	64.28 ± 2.25	69.30 ± 2.05	65.83 ± 1.42	0.034	0.402	0.690	0.123	0.204
Na+, mmol/L	146.5 ± 0.57	146.4 ± 0.47	146.8 ± 0.47	147.2 ± 0.47	147.8 ± 0.52	146.8 ± 0.35	0.229	0.578	0.389	0.808	0.611
K+, mmol/L^1^	4.45 ± 0.16	4.35 ± 0.15	4.50 ± 0.15	4.50 ± 0.15	4.23 ± 0.23	4.56 ± 0.16	0.465	0.597	0.612	0.244	0.990
Ca++, mmol/L	1.28 ± 0.01^a^	1.25 ± 0.01^ab^	1.24 ± 0.01^b^	1.26 ± 0.0^ab^	1.27 ± 0.01	1.25 ± 0.01	0.199	0.733	0.074	0.111	0.330
Cl-, mmol/L	103.0 ± 0.61	101.9 ± 0.52	102.6 ± 0.53	101.6 ± 0.52	102.4 ± 0.48	102.2 ± 0.46	0.916	0.090	0.851	0.846	0.561
Total carbon dioxide, mmol/L	34.95 ± 0.74	35.45 ± 0.62	35.86 ± 0.62	36.27 ± 0.62	35.23 ± 0.57	35.91 ± 0.39	0.127	0.419	0.781	0.285	0.521
Anion gap, mmol/L^1^	14.29 ± 0.35	14.53 ± 0.30	14.26 ± 0.31	14.36 ± 0.30	14.41 ± 0.28	14.34 ± 0.27	0.163	0.502	0.830	0.732	0.213
Hematocrit, %	33.24 ± 0.89^a^	34.68 ± 0.74^ab^	34.23 ± 0.74^ab^	35.65 ± 0.74^b^	35.12 ± 0.67	34.20 ± 0.47	0.113	0.080	0.860	0.322	0.604
Hemoglobin, g/L	113.1 ± 3.00^a^	118.1 ± 2.50^ab^	116.1 ± 2.49^ab^	121.2 ± 2.49^b^	119.3 ± 2.28	116.4 ± 1.59	0.122	0.066	0.868	0.347	0.593
Base excess (blood), mmol/L	9.76 ± 0.53^a^	10.14 ± 0.45^ab^	10.47 ± 0.44^ab^	11.26 ± 0.45^b^	10.08 ± 0.41	10.63 ± 0.28	0.044	0.102	0.556	0.249	0.566
Lactate, mmol/L^1^	2.34 ± 0.29	3.02 ± 0.24	3.11 ± 0.24	3.19 ± 0.24	2.65 ± 0.26	3.10 ± 0.16	0.089	0.209	0.203	0.120	0.671
Blood urea nitrogen, mg/Dl^2^	8.22 ± 0.68	7.06 ± 0.51	7.67 ± 0.54	6.69 ± 0.52	6.75 ± 0.55	7.67 ± 0.37	0.598	0.192	0.890	0.152	0.285
Creatinine, mg/dL	2.63 ± 0.10	2.59 ± 0.09	2.77 ± 0.09	2.61 ± 0.09	2.54 ± 0.08	2.71 ± 0.05	0.350	0.708	0.434	0.060	0.567
Calcium, mmol/L	2.65 ± 0.04	2.64 ± 0.03	2.59 ± 0.03	2.67 ± 0.03	2.67 ± 0.03	2.63 ± 0.02	0.846	0.420	0.389	0.552	0.351
Cholesterol, mmol/L	1.30 ± 0.06^a^	1.42 ± 0.05^ab^	1.32 ± 0.05^a^	1.49 ± 0.05^b^	1.48 ± 0.05^a^	1.35 ± 0.04^b^	0.329	0.022	0.999	0.020	0.168
Glucose, mmol/L^3^	5.23 ± 0.17^a^	5.82 ± 0.13^b^	5.33 ± 0.13^a^	5.16 ± 0.12^a^	5.43 ± 0.11	5.34 ± 0.08	0.113	0.157	0.002	0.809	0.191
Phosphate, mmol/L^3^	2.26 ± 0.10	2.16 ± 0.07	2.29 ± 0.08	2.29 ± 0.07	2.36 ± 0.07^a^	2.16 ± 0.05^b^	0.618	0.259	0.700	0.015	0.689
BHB, mmol/L	0.17 ± 0.05	0.11 ± 0.04	0.07 ± 0.04	0.09 ± 0.04	0.08 ± 0.04	0.12 ± 0.03	0.313	0.686	0.492	0.362	0.480
Magnesium, mmol/L^3^	0.73 ± 0.02	0.72 ± 0.02	0.72 ± 0.02	0.68 ± 0.02	0.70 ± 0.02	0.71 ± 0.01	0.321	0.144	0.561	0.762	0.435
NEFA, mmol/L	0.33 ± 0.08	0.38 ± 0.07	0.33 ± 0.07	0.35 ± 0.07	0.30 ± 0.06	0.37 ± 0.04	0.753	0.865	0.975	0.384	0.530
Insulin, μ IU/mL^4^	2.34 ± 0.19^a^	3.16 ± 0.15^b^	2.97 ± 0.16^b^	2.80 ± 0.16^ab^	2.82 ± 0.15	2.87 ± 0.11	0.256	0.053	0.006	0.810	0.655
Leptin, ng/mL^1^	6.31 ± 0.75^a^	7.59 ± 0.63^ab^	8.19 ± 0.64^ab^	8.62 ± 0.64^b^	7.72 ± 0.65	7.76 ± 0.45	0.066	0.173	0.476	0.953	0.782
Osteocalcin, ng/mL	78.5 ± 6.19^a^	78.4 ± 5.31^a^	64.9 ± 5.44^ab^	60.6 ± 5.57^b^	76.6 ± 4.96	67.4 ± 3.40	0.019	0.812	0.532	0.490	0.379

^1^Covariable was days on transition diet (*P* < 0.05).

^2^Covariables were breed of sow and weight at farrowing house entry (*P* < 0.05).

^3^Breed of sow (*P* < 0.05).

^4^Log transformed.

^a,b^Different superscripts within a row and within treatment indicate pairwise comparisons at *P* < 0.05.

The marked increase in osteocalcin (76.55 vs. 67.40 ng/mL) indicates the potential for negative DCAD diets to influence energy metabolism through skeletal hormonal metabolism similar to that in dairy cows. Lean et al. (2014) found that negative DCAD diets act to stimulate bone turnover, acidify the blood, and therefore potentially enhance the vitamin K-mediated decarboxylation of osteocalcin to the uncarboxylated form of osteocalcin. This may then lead to enhanced effects on metabolism. Further detailed metabolic studies in the sow would elucidate these aspects of metabolism. Differences among the four treatment groups at farrowing in glucose, cholesterol, and insulin concentrations (Table 8), provide more evidence that differences in dietary DCAD and/or vitamin D source may influence energy metabolism at the time of parturition. There is considerable interest in associations between bone metabolism and energy and protein metabolism across a range of species (Lean et al., 2014). Serum osteocalcin is a marker for bone formation and can therefore provide information on bone turnover, calcium metabolism, and sow metabolic state (Ordaz et al., 2022). There are few published data on osteocalcin concentrations in sows throughout their breeding cycle, however, a recent review aimed to characterize osteocalcin in breeding sows and found that osteocalcin levels reduce at farrowing and then increase into lactation (Ordaz et al. 2022), supporting results found in the current study. Low osteocalcin concentrations at farrowing indicate mineral mobilization from the skeleton, in conjunction with placental calcium transfer towards calcium-rich milk production. All other blood parameters were similar between negative and positive DCAD rations.

Vitamin D type (calcidiol added to cholecalciferol) had few effects on blood parameters at day 1 post-parturition (Table 8); however, sows that received calcidiol in their transition ration had lower oxygen partial pressure and higher cholesterol concentration than sows that received no calcidiol (*P* < 0.05) and log-transformed insulin tended (*P* = 0.05) to be higher in calcidiol fed sows. There were few significant interactions between DCAD and calcidiol on blood parameters on day 1 post-parturition, except that negative DCAD + CA-fed sows had higher glucose concentration than sows in the other three transition groups and sows fed the negative DCAD + no CA diet had lower insulin concentration than all other groups, and sows in the negative DCAD + CA treatment group had higher insulin concentration than other groups (Table 8). There were also few DCAD × calcidiol × parity interactions, except for oxygen partial pressure. Primiparous sows fed the negative DCAD + no CA diet had higher oxygen partial pressure (53.83 ± 4.50 mmHg) than all other groups (primiparous negative DCAD + CA, 33.86 ± 2.30; primiparous positive DCAD + no CA, 35.16 ± 2.94, primiparous positive DCAD + CA, 37.60 ± 2.40; multiparous negative DCAD + no CA, 35.29 ± 3.14; multiparous negative DCAD + CA, 33.85 ± 2.30; multiparous positive DCAD + no CA, 33.54 ± 2.94; and multiparous positive DCAD + CA, 32.33 ± 2.40 mmHg; *P* < 0.05).

Blood measures 21 d post-parturition are presented in Table 9. There were no effects of DCAD on any blood parameter; however, sows that received calcidiol had higher P (1.78 ± 0.03) than sows fed cholecalciferol (1.70 ± 0.03) in their transition ration (*P* < 0.05). All blood parameters were similar between parity groups and no interactions between DCAD, calcidiol, or parity for any blood parameter occurred on day 21 post-parturition.

**Table 9.** Blood metabolite measures on day 21 post-parturition of sows (*n* = 74) that received either a negative or positive DCAD transition diet and with or without calcidiol (CA). Values for treatments are marginal means ± SE. Covariables tested were weight at farrowing house entry, days on transition diet, and breed of sow

Item	Negative DCAD		Positive DCAD		Parity		*P* value				
	No CA	CA	No CA	CA	Primiparous	Multiparous	DCAD	Calcidiol	DCAD × calcidiol	Parity	DCAD × calcidiol × Parity
Calcium, mmol/L	2.60 ± 0.03	2.63 ± 0.02	2.64 ± 0.02	2.66 ± 0.02	2.62 ± 0.02	2.65 ± 0.01	0.069	0.267	0.954	0.348	0.372
Cholesterol, mmol/L	2.19 ± 0.11	2.15 ± 0.09	2.26 ± 0.09	2.37 ± 0.10	2.17 ± 0.08	2.28 ± 0.07	0.177	0.746	0.664	0.271	0.303
Glucose, mmol/L	5.63 ± 0.18	5.52 ± 0.16	5.44 ± 0.16	5.52 ± 0.17	5.59 ± 0.15	5.49 ± 0.10	0.912	0.479	0.938	0.832	0.192
Phosphate, mmol/L^1^	1.69 ± 0.04^a^	1.72 ± 0.04^ab^	1.69 ± 0.04^a^	1.83 ± 0.04^b^	1.68 ± 0.04	1.76 ± 0.03	0.104	0.043	0.462	0.363	0.157
BHB, mmol/L	0.04 ± 0.01	0.05 ± 0.01	0.05 ± 0.01	0.05 ± 0.01	0.03 ± 0.01	0.05 ± 0.00	0.326	0.808	0.261	0.090	0.573
Magnesium, mmol/L	0.86 ± 0.02	0.83 ± 0.02	0.84 ± 0.02	0.84 ± 0.02	0.85 ± 0.02	0.84 ± 0.02	0.952	0.428	0.288	0.279	0.613
NEFA, mmol/L	0.13 ± 0.04	0.22 ± 0.03	0.16 ± 0.04	0.18 ± 0.04	0.17 ± 0.03	0.18 ± 0.02	0.661	0.193	0.477	0.702	0.868
Insulin, μIU/mL^2^	3.52 ± 0.21	3.45 ± 0.18	3.64 ± 0.19	3.50 ± 0.20	3.64 ± 0.18	3.47 ± 0.12	0.905	0.271	0.706	0.389	0.119
Leptin, ng/mL	10.07 ± 0.93	10.27 ± 0.82	10.35 ± 0.86	9.49 ± 0.91	9.28 ± 1.12	10.43 ± 0.72	0.706	0.954	0.767	0.390	0.242
Osteocalcin, ng/mL	111.9 ± 9.01	110.7 ± 7.68	110.9 ± 8.36	104.4 ± 8.78	109.3 ± 8.67	109.6 ± 5.71	0.926	0.970	0.594	0.950	0.494

^1^Covariable was breed of sow (*P <* 0.05).

^2^Log transformed.

^a,b^Different superscripts within a row and within treatment indicate pairwise comparisons at *P* < 0.05.

### Colostrum and milk composition

The main effects of DCAD or calcidiol did not influence milk or colostrum composition (Table 10) and there were no interactions between DCAD and calcidiol. There were, however, DCAD × calcidiol × parity interactions. Primiparous sows fed the negative DCAD + no CA diet had higher milk fat (3.89 ± 0.68%) than primiparous sows that had a negative DCAD + CA diet (2.04 ± 0.39%) or multiparous sows fed the positive DCAD + CA diet (2.06 ± 0.42%), multiparous sows fed the positive DCAD + no CA ration (3.65 ± 0.50 %), also had higher milk fat percentage than those two groups. Primiparous sows fed the negative DCAD + CA or the positive DCAD + no CA diet had lower percentage of total milk solids (13.91 ± 0.68% and 13.95 ± 0.99%, respectively) than all other diets. Primiparous sows tended to have higher fat content than multiparous sows (Table 10; *P* = 0.07) which is a finding similar to that of Pedersen et al. (2020), but was not reflected in higher litter weights at day 21 of lactation. No other differences were observed between the two parity groups. The lack of difference in piglet weights was consistent with a lack of difference in colostrum or milk fat or protein content (Table 4).

**Table 10.** Colostrum and milk parameters of sows that received either a negative or positive DCAD transition diet and with or without calcidiol (CA). Values for treatments are marginal means ± SE. Covariables tested were weight at farrowing house entry, days on transition diet, and breed of sow

Item		Negative DCAD		Positive DCAD		Parity		*P* value				
	*n*	No CA	CA	No CA	CA	Primiparous	Multiparous	DCAD	Calcidiol	DCAD × calcidiol	Parity	DCAD × calcidiol × parity
Colostrum solids, %	86	26.23 ± 0.58	25.71 ± 0.56	25.88 ± 0.60	25.65 ± 0.54	25.78 ± 0.52	25.88 ± 0.34	0.582	0.762	0.773	0.814	0.867
Colostrum IgG, mg/mL^1^	86	70.90 ± 4.36	62.84 ± 4.40	63.82 ± 4.59	73.35 ± 4.40	66.70 ± 4.74	68.37 ± 3.00	0.778	0.844	0.193	0.666	0.176
Milk fat, %^1^	68	2.71 ± 0.35	2.92 ± 0.30	2.52 ± 0.30	2.63 ± 0.32	3.16 ± 0.34	2.27 ± 0.22	0.494	0.713	0.521	0.067	0.003
Milk protein, %	68	7.67 ± 0.40	8.46 ± 0.41	8.15 ± 0.36	8.23 ± 0.40	8.44 ± 0.34	7.95 ± 0.24	0.742	0.338	0.670	0.241	0.189
Milk somatic cell^2^	72	7.12 ± 0.29	6.57 ± 0.27	6.73 ± 0.26	6.91 ± 0.29	6.51 ± 0.32	6.70 ± 0.21	0.817	0.404	0.177	0.653	0.031
Milk urea^3^	71	49.28 ± 6.97	39.11 ± 6.59	46.65 ± 6.09	41.39 ± 6.88	43.20 ± 5.70	46.14 ± 4.10	0.877	0.126	0.952	0.660	0.803
Milk lactose, %	71	4.99 ± 0.14	4.79 ± 0.14	4.74 ± 0.12	4.83 ± 0.14	4.50 ± 0.12	4.75 ± 0.08	0.462	0.450	0.221	0.085	0.511
Milk solids, %	70	14.83 ± 0.61	15.25 ± 0.59	15.48 ± 0.55	15.63 ± 0.62	15.67 ± 0.53	15.11 ± 0.37	0.340	0.870	0.508	0.680	0.005

^1^Covariable was farrow house entry weight (*P <* 0.05).

^2^Log transformed.

^3^Days on transition diet (*P <* 0.05).

^a,b^Different superscripts within a row and within treatment indicate pairwise comparisons at *P* < 0.05.

There was no evidence in this study of the increased milk production responses seen in cows (Lean et al., 2014, 2019; Santos et al., 2019). It is important to note that we sampled milk after weaning when all sows had been on the lactating sow ration for in excess of 20 d which may explain why no differences in milk composition were observed. Guo et al. (2019) also found that lowering the DCAD of the diet during late gestation and lactation did not alter fat, lactose, protein, and solids in sow milk at day 1 or day 18 postpartum. However, calcidiol supplementation during lactation increased the solids, protein, and lactose content of milk at day 14 (Zhou et al., 2017) and increased colostrum fat content, and improved mRNA and protein levels of genes that regulate milk fat synthesis (Wang et al., 2020). Despite these differences in milk composition, both studies had an increase in piglet weight gain which may reflect diets that were continued throughout lactation. In the current study, all sows were fed a standard lactating sow ration on day 4 of lactation and remained on this diet until weaning. The lactating sow ration had a high DCAD value (178 mEq/kg, Table 2), and no calcidiol was included. It would be worthwhile continuing the transition diets into lactation further to determine if this would influence milk production, piglet weight gain, and survival.

### Health and subsequent reproduction

There were 10 sow mortalities in this study; negative DCAD + no CA, four sows died (two caesarian sections, one liver torsion, and one euthanized due to lameness); negative DCAD + CA, two sows died (two euthanized for broken leg and lameness); positive DCAD + no CA, two sows died (one euthanized due to abortion, and one liver torsion); and positive DCAD + CA, two sows died (two euthanized due to lameness and injury). The odds of health disorders were not impacted by the DCAD of the transition diet, calcidiol, parity, or their interactions (Table 11). All sows had similar incidences of mastitis (13.7%), udder edema (5.1%), engorged udder (10.0%), retained piglets and/or placenta (2.6%), and vaginal discharge (14.7%). The DCAD, calcidiol, and parity had no effect on percentage mated, median days to mating, culling or dead, percentage of dead sows, and conception or pregnancy percentages postweaning (Table 12). While disease did not differ among treatments the statistical power was low to detect differences. For example, there was a substantially lesser incidence of vaginal discharge in primiparous (9.10%) than in multiparous sows (17.2%), but this did not approach significance (Table 11).

**Table 11.** Percentage, odds ratio (**OR**) and significant risk of clinical health disorders for sows (*n* = 328) fed either a negative or positive DCAD transition diet and with or without calcidiol (CA). Percentages are raw means. Covariables tested included variables tested were weight at farrowing house entry, days on transition diet, and breed of sow and breed of boar

Disorder	Treatment (%)				Parity (%)		Treatment OR^1^ (SE)			
	Negative DCAD + no CA	Negative DCAD + CA	Positive DCAD + no CA	Positive DCAD + CA	Primiparous	Multiparous	Negative DCAD + CA^1^	Positive DCAD	Positive DCAD + CA	Parity^2^
Mastitis	14.3	13.9	12.3	14.1	11.4	14.7	0.973 (0.470)	0.840 (0.415)	0.981 (0.487)	1.34 (0.529)
Oedema^3^	7.14	4.17	2.74	6.25	6.82	4.19	0.531 (0.405)	0.273 (0.239)	0.730 (0.519)	0.164 (0.138)
Engorged udder	8.57	8.33	13.69	9.38	10.23	9.95	1.000 (0.614)	1.831 (1.057)	1.175 (0.737)	0.932 (0.413)
Retained^4^	4.29	1.39	0	4.69	2.27	2.62	–	–	–	1.169 (0.990)
Discharge^3^	15.7	13.9	15.1	14.1	9.10	17.2	0.788 (0.378)	0.849 (0.411)	0.781 (0.391)	0.933 (0.481)

^1^Referent is negative DCAD.

^2^Referent is primiparous.

^3^Covariable was farrowing house entry weight.

^4^Interaction of DCAD and calcidiol were not calculated because of zero in the positive DCAD treatment (DCAD OR = 0.781 ± 0.604 and calcidiol OR = 1.404 ± 1.087).

**Table 12.** Percentage, odds ratio and SE in brackets of sows (*n* = 328) being mated within 7 d of weaning, days to removal, the percent of sows that died during the experiment, and subsequent reproduction (pregnancy and conception rates) for either a negative or positive DCAD transition diet and with or without calcidiol (CA). Percentages per treatment and parity are estimated marginal means. Covariables tested included weight at farrowing house entry, days on transition diet, breed of sow, and breed of boar

Item	Treatment				Parity		Odds ratio (SE)			
	Negative DCAD + no CA	Negative DCAD + CA	Positive DCAD + no CA	Positive DCAD + CA	Primiparous	Multiparous	Negative DCAD + CA^1^	Positive DCAD	Positive DCAD + CA	Parity^2^
Mated within 7 d, %^3^	87.1	90.0	88.9	93.1	84.3	92.4	1.335 (0.750)	1.196 (0.673)	1.774 (1.162)	2.217 (0.941)^4^
Median days to mated or removed	5.00	5.00	5.00	5.00	5.00	5.00	1.07 (0.683)	1.05 (0.786)	1.16 (0.432)	1.02 (0.847)
Cull or dead, %^5^	26.2	16.7	21.3	26.6	16.2	25.4	0.546 (0.222)	0.652 (0.265)	0.907 (0.355)	1.128 (0.466)
Sow death, %	4.76	2.38	2.50	2.53	3.03	3.07	0.479 (0.429)	0.513 (0.452)	0.520 (0.458)	1.009 (0.709)
Conception, %	93.6	98.6	98.4	96.6	100.0	95.3	4.847 (24.75)	4.352 (21.52)	1.952 (5.936)	—
Pregnant, %	91.6	97.2	96.8	92.7	98.8	92.9	3.321 (2.876)	2.837 (2.472)	1.172 (0.839)	0.139 (0.147)

^1^Referent is negative DCAD + no CA.

^2^Referent is primiparous.

^3^Covariable was breed of boar.

^4^ *P <* 0.05.

^5^Covariable was weight at farrowing house entry.

—, not tested in the model due to 100% conception in primiparous sows.

In the subsequent litter, the main effect of DCAD affected the number of total born piglets sows (positive DCAD, 14.7 ± 0.3 and negative DCAD, 13.8 ± 0.3 piglets; *P* = 0.029), but the main effect of calcidiol supplementation did not influence results (14.1 ± 0.3 and 14.4 ± 0.3 piglets, respectively; *P* = 0.869). Similarly, parity did not impact the subsequent number of total piglets born; however, there were significant DCAD × calcidiol × parity interactions (Table 13, *P* = 0.002). Multiparous sows fed the negative DCAD + CA diet had less piglets born in the subsequent litter (12.8 ± 0.5) compared to all other groups except the primiparous sows fed the negative DCAD + no CA diet (12.8 ± 0.7). The latter differed from all others except the multiparous positive DCAD + CA group (14.3 ± 0.5) and primiparous sows fed the negative DCAD + CA ration (14.7 ± 0.7). Primiparous sows fed the positive DCAD + no CA (15.6 ± 0.7), primiparous sows fed the negative DCAD + CA (15.0 ± 0.8), multiparous sows fed a negative DCAD + no CA (14.8 ± 0.6), and multiparous sows fed the positive DCAD + no CA (14.7 ± 0.6) had a similar number of piglets born to all but the lowest two groups (multiparous negative DCAD + CA, and, primiparous negative DCAD + no CA).

**Table 13.** The total number of born piglets, piglets born alive in the subsequent gestation for sows (*n* = 236) that received either a negative or positive DCAD transition diet and with or without calcidiol (CA). Values for treatments are marginal means ± SE. Covariables tested included days on transition diet, breed of sow, and breed of boar

Item	Negative DCAD		Positive DCAD		Parity		*P* value				
	No CA	CA	No CA	CA	Primiparous	Multiparous	DCAD	Calcidiol	DCAD × calcidiol	Parity	DCAD × calcidiol × parity
Total born, *n*	14.07 ± 0.52^ab^	13.52 ± 0.41^a^	14.78 ± 0.44^ab^	14.69 ± 0.46^b^	14.56 ± 0.37^a^	14.07 ± 0.31^b^	0.029	0.869	0.638	0.392	0.002^1^
Born alive, *n*	12.76 ± 0.43^ab^	12.16 ± 0.40^a^	13.23 ± 0.42^ab^	13.37 ± 0.45^b^	13.51 ± 0.31^a^	12.73 ± 0.31^b^	0.0213	0.886	0.895	0.028	0.019

^1^Covariate was breed of sow (*P* < 0.05).

Overall, sows that received positive DCAD diets had more piglets born alive in the subsequent litter compared to sows that received negative DCAD diets (13.3 ± 0.3 and 12.5 ± 0.3 piglets, respectively; *P* = 0.021). The inclusion of calcidiol did not affect the subsequent number of piglets born alive; however, the main effect of parity and interactions of DCAD × calcidiol × parity did (*P* = 0.028 and 0.019, respectively, Table 13). Multiparous sows fed the negative DCAD + CA diet (11.5 ± 0.5) had less live piglets than primiparous sows fed the negative DCAD + no CA diet (12.0 ± 0.7 piglets) and the positive DCAD + no CA diet (12.5 ± 0.5 piglets). The primiparous sows fed the positive DCAD + no CA diet (14.5 ± 0.7 piglets) had more live piglets than the multiparous sows fed the positive DCAD + no CA diet. The former did not differ from the multiparous negative DCAD + no CA (13.2 ± 0.5 piglets), primiparous DCAD + CA (13.5 ± 0.7 piglets), primiparous positive DCAD + CA (14.1 ± 0.7 piglets) and multiparous positive DCAD + CA (13.0 ± 0.6 piglets) groups.

These responses differ from Henman et al. (2023) who found similar numbers of piglets born to sows in the subsequent gestation, but a reduction in the number of stillborn piglets resulting in an average of an additional 0.5 piglets for the higher rate of acidogenic protein meal inclusion. The acidogenic protein meal was fed during lactation as well as the prior prefarrowing interval in that study. When the four treatment groups in this study, that were metabolically acidified, were compared to sows fed a non-acidogenic lactation ration (178 mEq/kg) during the transition period, there were less stillbirths for the negative DCAD + CA (−2.1 mEq/kg) and positive DCAD (68.4 mEq/kg) treatment groups, compared to sows fed the lactating sow ration (Weaver et al., 2023). There was also a tendency for more than 0.9 additional piglets to be born in the subsequent litter for the negative DCAD and both positive DCAD groups compared to sows fed a more positive DCAD lactating sow diet (Weaver et al., 2023). The risk of stillbirth and mummification did not differ among groups (Table 14), but was markedly increased for older sows (*P* < 0.05).

**Table 14.** Mean number per sow and SD in brackets by treatment and parity and incidence (IRR) with SE in brackets of stillborn piglets and mummies in the subsequent litter for either a negative or positive DCAD transition diet and with or without calcidiol (CA). The IRR were estimated using negative binomial analysis with sow as a random effect. Covariables tested included weight at farrowing house entry, days on transition diet, breed of sow, and breed of boar

Item	Treatment				Parity		IRR (SE)			
	Negative DCAD + no CA	Negative DCAD + CA	Positive DCAD + noCA	Positive DCAD + CA	Primiparous	Multiparous	Negative DCAD + CA^1^	Positive DCAD + no CA	Positive DCAD + CA	Parity^2^
Subsequent stillborn/litter	1.281 (1.66)	1.353 (2.06)	1.627 (1.89)	1.269 (1.69)	1.024 (1.431)	1.578 (2.00)	1.166 (0.257)	1.208 (0.289)	0.933 (0.241)	1.569^3^ (0.270)
Subsequent mummified	0.509 (0.826)	0.603 (0.917)	0.458 (0.729)	0.442 (0.698)	0.427 (0.817)	0.552 (0.792)	1.976 (0.721)	1.352 (0.516)	1.175 (0.476)	1.555^3^ (0.383)

^1^Referent is Negative DCAD + no CA.

^2^Referent is primiparous.

^3^Parity significant (*P <* 0.05).

## Conclusion

The most notable responses in this study are the reduction in litters with stillbirths for the negative DCAD + CA sows and the higher born alive for the positive DCAD sows in the subsequent lactation. The former results are similar to earlier studies evaluating responses to a negative DCAD diet in pigs. The negative diet, DCAD + CA led to a 28% reduction in odds of stillbirth compared to that of sows on the negative DCAD + no CA diet and an even greater reduction in odds compared to that of sows on the positive DCAD + CA diet. However, there was a reduction in piglets born and born alive for negative DCAD diets in the subsequent farrowing. This result may reflect a quite marked difference in metabolic acidosis to the two positive DCAD groups as reflected in the urinary pH and blood chemistry at farrowing. As noted, the positive DCAD group also had metabolic acidosis as indicated by the urinary pH, indicating a need to understand the effects of carbohydrates in diets on metabolic acidosis. The optimal acidification of sows prefarrowing needs to be determined; however, the results of this study and others indicate potential benefits of metabolic acidification in the sow on piglet survival and support the findings demonstrating the role of skeletal metabolism in the energy metabolism of mammals.

## Supplementary Material

skae027_suppl_Supplementary_Tables_S1

## Abbreviations

BHB: 3-hydroxybutyrate
CA: calcidiol: a vitamin D metabolite 25-OH-D_3_
DCAD: dietary anion cation difference as mEq/kg of (Na + K) − (Cl + S)
dEB: dietary electrolyte balance
IgG: immunoglobulin G
NEFA: nonesterified free fatty acids
RFID: radio frequency identification
RIA: radioimmunoassay
