# Supplementary material for: Effects of negative dietary cation–anion difference and calcidiol supplementation in transition diets fed to sows on piglet survival, piglet weight, and sow metabolism
Source: J Anim Sci. 2024 Jan 29;102:skae027. doi: 10.1093/jas/skae027 (PMC10889728; doi:10.1093/jas/skae027)
Supplement: skae027_suppl_Supplementary_Tables_S1 [file skae027_suppl_supplementary_tables_s1.docx]

**Supplementary Table 1.** Backfat measures at P2 for sows that received either a negative or positive DCAD transition diet and calcidiol (CA). Values for treatments are marginal means ± SE.

| **Item** | **Negative DCAD** | | **Positive DCAD** | | **Parity** | | ***P*-value** | | | | |
| --- | --- | --- | --- | --- | --- | --- | --- | --- | --- | --- | --- |
|  | **No CA** | **CA** | **No CA** | **CA** | **Primiparous** | **Multiparous** | **DCAD** | **Calcidiol** | **DCAD × calcidiol** | **Parity** | **DCAD × calcidiol × parity** |
| P2 backfat, mm |  |  |  |  |  |  |  |  |  |  |  |
| FH entry | 28.9 ± 0.6 | 27.6 ± 0.6 | 28.5 ± 0.6 | 28.2 ± 0.7 | 27.8 ± 0.6 | 28.5 ± 0.4 | 0.779 | 0.279 | 0.496 | 0.294 | 0.980 |
| Day 1 | 27.4 ± 0.6 | 27.7 ± 0.6 | 27.2 ± 0.6 | 27.7 ± 0.6 | 28.9 ± 0.6^a^ | 26.9 ± 0.4^b^ | 0.799 | 0.647 | 0.685 | 0.012 | 0.568 |
| Day 21 | 21.6 ± 0.7 | 23.0 ± 0.6 | 22.5 ± 0.7 | 22.6 ± 0.7 | 23.8 ± 0.7^a^ | 21.9 ± 0.5^b^ | 0.744 | 0.414 | 0.345 | 0.023 | 0.863 |
|  |  |  |  |  |  |  |  |  |  |  |  |
| P2 backfat change, mm |  |  |  |  |  |  |  |  |  |  |  |
| FH entry to d 1 | -0.8 ± 0.4 | -0.3 ± 0.4 | -1.4 ± 0.4 | -1.0 ± 0.4 | -0.9 ± 0.4 | -0.9 ± 0.3 | 0.208 | 0.268 | 0.862 | 0.976 | 0.418 |
| Day 1 to 21 | -4.9 ± 0.5 | -4.4 ± 0.5 | -4.3 ± 0.5 | -5.1 ± 0.5 | -5.3 ± 0.5 | -4.4 ± 0.3 | 0.765 | 0.884 | 0.071 | 0.155 | 0.174 |
| FH entry to d 21 | -6.3 ± 0.5 | -5.0 ± 0.4 | -5.6 ± 0.5 | -5.9 ± 0.5 | -6.3 ± 0.5 | -5.4 ± 0.3 | 0.452 | 0.240 | 0.055 | 0.111 | 0.325 |
| FH entry to weaning | -4.9 ± 0.5 | -4.4 ± 0.5 | -4.3 ± 0.5 | -5.1 ± 0.5 | -5.3 ± 0.5 | -4.4 ± 0.3 | 0.765 | 0.884 | 0.071 | 0.155 | 0.174 |
| Day 1 to weaning | -6.1 ± 0.5 | -5.7 ± 0.5 | -6.1 ± 0.5 | -6.2 ± 0.5 | -6.5 ± 0.5 | -5.8 ± 0.3 | 0.692 | 0.501 | 0.228 | 0.358 | 0.078 |

FH = farrow house.

^ab^ Different superscripts within a row and within treatment indicate pairwise comparisons at *P* < 0.05.
